# Immunomodulatory Effect of Hypericin-Mediated Photodynamic Therapy on Oral Cancer Cells

**DOI:** 10.3390/pharmaceutics16010042

**Published:** 2023-12-27

**Authors:** Marcin Olek, Agnieszka Machorowska-Pieniążek, Zenon P. Czuba, Grzegorz Cieślar, Aleksandra Kawczyk-Krupka

**Affiliations:** 1 Doctoral School of Medical University of Silesia, 40-055 Katowice, Poland; 2 Department of Orthodontics, Faculty of Medical Sciences in Zabrze, Medical University of Silesia, 40-055 Katowice, Poland; apieniazek@sum.edu.pl; 3 Department of Microbiology and Immunology, Faculty of Medical Sciences in Zabrze, Medical University of Silesia, 40-055 Katowice, Poland; zczuba@sum.edu.pl; 4 Department of Internal Diseases, Angiology and Physical Medicine, Center for Laser Diagnostics and Therapy, Faculty of Medical Sciences in Zabrze, Medical University of Silesia, 40-055 Katowice, Poland; cieslar1@tlen.pl

**Keywords:** photodynamic therapy, oral cancer, hypericin, immunomodulatory effect

## Abstract

In 2020, there were 377,713 new oral and lip cancer diagnoses and 177,757 deaths. Oral cancer is a malignancy of the head and neck region, and 90% of cases are squamous cell carcinomas (OSCCs). One of the alternative methods of treating pre-cancerous lesions and oral cancer is photodynamic therapy (PDT). In addition to the cytotoxic effect, an important mechanism of PDT action is the immunomodulatory effect. This study used the OSCC (SCC-25) cell line and the healthy gingival fibroblast (HGF-1) line. A compound of natural origin—hypericin (HY)—was used as the photosensitizer (PS). The HY concentrations of 0–1 µM were used. After two hours of incubation with PS, the cells were irradiated with light doses of 0–20 J/cm^2^. The MTT test determined sublethal doses of PDT. Cell supernatants subjected to sublethal PDT were assessed for interleukin 6 (IL-6), soluble IL-6 receptor alpha (sIL-6Ralfa), sIL-6Rbeta, IL-8, IL-10, IL-11 IL-20, IL-32, and Pentraxin-3 using the Bio-Plex Pro^TM^ Assay. The phototoxic effect was observed starting with a light dose of 5 J/cm^2^ and amplified with increasing HY concentration and a light dose. HY-PDT affected the SCC-25 cell secretion of sIL-6Rbeta, IL-20, and Pentraxin-3. HY alone increased IL-8 secretion. In the case of HGF-1, the effect of HY-PDT on the secretion of IL-8 and IL-32 was found.

## 1. Introduction

Photodynamic therapy (PDT) is a method of treatment in which a photosensitizer (PS) is applied to the desired place, which is then activated by light of the appropriate wavelength and dose. In the presence of oxygen in the tissues, a photodynamic reaction (PDR) occurs [1]. After irradiation, according to the absorption spectrum of PS located inside the cell, a transition occurs from the basic singlet state to the excited singlet state by cause of the absorption of photon energy. Some of the energy is radiated in the form of a fluorescence quantum. The therapeutic form of PS is the excited triplet state, which is formed because of a non-radiative inter-combination transition [2]. As a result of the reaction of the excited photosensitizer with environmental biomolecules, free radicals and anion radicals of the photosensitizer and the substrate are generated. The electrons interact with oxygen to form reactive oxygen species (ROS). This process results in oxidative stress and the death of cancer cells, which is a type I of the PDT mechanism. Type II photodynamic reaction involves a direct transfer of energy from the triplet excited form of PS to an oxygen molecule, resulting in the formation of singlet oxygen, which has a powerful oxidizing effect. It is believed that the most important type of PDT reaction is type II [3]. The percentage of mechanisms involved in the photodynamic reaction depends on many factors, such as the concentration of oxygen in the tissues, the type of photosensitizer, and the pH of the environment. PDT leads to programmed cell death, necrosis, or autophagy, depending on the intracellular location of PS and damage to given cellular structures [4]. PDR also occurs in the blood vessels supplying the tumor, disrupting the vessel walls, aggregating platelets, and, consequently, causing a loss of blood supply to the tumor and its necrosis. A significant role of PDT is the activation of the immune system. Neutrophils and macrophages are activated due to the release of cytokines, inflammatory mediators, and proteins. After phagocytosis of damaged cells, macrophages present antigens to helper CD4 T cells, which activate CD8 cytotoxic T cells [1,5].

In 2020, there were 377,713 new oral and lip cancer diagnoses and 177,757 deaths [6]. Oral cancer is a malignancy of the head and neck region, and 90% of cases are squamous cell carcinomas (OSCCs). Predisposing factors include smoking, chewing betel leaves, alcohol consumption, human papillomavirus infection, and poor oral hygiene [7,8,9]. The standard treatment method for OSCC is the excision of the tumor within the healthy tissues, which is accompanied by surgery of the lymphatic system of the neck in the case of metastases to the lymph nodes [10]. Adjuvant radiotherapy or chemotherapy is also used depending on the cancer stage and the surgery’s radicality [11]. As a result of typical complications after traditional treatment, patients’ quality of life decreases [12]. The most frequently observed consequences of the surgical procedure are speech, chewing, and swallowing disorders and aesthetic problems [13]. Radiotherapy is also not indifferent, causing xerostomia, increased susceptibility to caries and periodontitis, tissue fibrosis, and opportunistic infections [14,15]. Because of the above, alternative treatment methods are sought, and PDT is one of them. The use of PDT in precancerous lesions and OSCC is being tested in preclinical and clinical trials with encouraging results [16,17,18,19]. One of the important directions of research is to understand the immunomodulatory effect of PDT.

The aim of our study was to evaluate the effect of PDT on the secretory activity of persistent oral squamous cell carcinoma cells and healthy cells that occur in the tumor environment fibroblasts.

## 2. Materials and Methods

### 2.1. Chemicals

Hypericin (HY), dimethyl sulfoxide (DMSO), MTT (3-[4,5-dimethylthiazol-2-yl]-2,5-diphenyltetrazolium bromide), and hydrocortisone were purchased from Sigma-Aldrich (St. Louis, MO, USA). Dulbecco’s Modified Eagle’s Medium (DMEM), DMEM: F-12, inactivated fetal bovine serum (FBS), and trypsin (0.23%)-ethylenediaminetetraacetic acid (EDTA) (0.53 mM) were obtained from ATCC (Manassas, VA, USA). Dulbecco’s phosphate-buffered saline (DPBS) without calcium and magnesium ions was obtained from PAA. Bio-Plex Pro^TM^ Assays were obtained from BIO-RAD Laboratories, Inc. (Hercules, CA, USA). All other chemicals were of analytical grade or purer.

### 2.2. Cell Cultures

Two cell lines were used in this study. The first one is an OSCC obtained from the tongue of a 70-year-old man—SCC-25 (ATCC CRL-1628). The second cell line is healthy gingival fibroblasts from a 28-year-old man—HGF-1 (ATCC CRL-2014). Frozen cell lines were purchased from ATCC (American Type Cell Culture—ATCC LGC Limited, London, UK).

### 2.3. Preparation of Cell Cultures

After defrosting, the cells were cultured strictly according to the manufacturer’s recommendations. DMEM: F12 supplemented with 10% FBS and 400 ng/mL hydrocortisone was used to culture the SCC-25 line. For HGF-1, the culture medium was DMEM supplemented with 10% FBS. Cultures were carried in culture flasks with 25 cm^2^ and 75 cm^2^ cultivation areas at 37 °C, 5% carbon dioxide, and constant humidity. Culture media were changed 2–3 times a week for both lines. Cell passage was performed when the monolayer did not exceed 80% confluency. A 0.25% trypsin solution with 0.53 mM EDTA was used to harvest the cells. After 5–10 min of trypsinization, the cells were diluted in a culture medium, centrifuged, and resuspended in a culture medium.

### 2.4. Incubation of Cells with the HY

A 1 mM HY stock solution in DMSO was diluted to final concentrations of 0.25 µM, 0.5 µM, and 1 µM in the appropriate culture medium. The final DMSO concentration did not exceed 0.1%. The cells were suspended in appropriate culture media, HGF in the amount of 1 × 10^4^/mL, and SCC-25 in the amount of 1 × 10^5^/mL. After the cells were seeded on a 96-well plate at 200 µL per well, incubation was carried out for 24 h to obtain adherence. The medium was changed, and HY was added at concentrations of 0 µM, 0.25 µM, 0.5 µM, and 1 µM. After 2 h incubation, the medium was changed again, and the cells were washed with PBS without calcium and magnesium ions to remove unabsorbed PS.

### 2.5. Fluorescence Microscopy

The presence of HY in the cells was confirmed using the Olympus IX51 (Olympus Inc., Tokyo, Japan) with a color view camera and Cell F version 2.6 software (Soft Imaging System GmbH, Münster, Germany).

### 2.6. Cell Exposure to Light

The cells were irradiated with a PDT TP1 photodynamic therapy lamp (Cosmedico Medizintechnik GmbH, Stuttgart, Germany). It is an incoherent light source that emits light with wavelengths of 580–720 nm after installing an orange and infrared filter. To avoid the thermal effect, a double water filter was used. With the water filters, the fluence rate was 35 mW/cm^2^. Cells were exposed to light at doses of 0 J/cm^2^, 1 J/cm^2^, 2 J/cm^2^, 5 J/cm^2^, 10 J/cm^2^, and 20 J/cm^2^. The exposure time was calculated automatically by the lamp controller. After irradiation, the cells were incubated for 24 h under the same conditions described previously.

### 2.7. MTT Assay for Cytotoxicity Evaluation

Supernatants were gently collected from above the cell surface. The cells were washed twice with PBS without magnesium and calcium ions. The appropriate culture medium with 0.5 mg/mL MTT was added to the cells. Only living cells can metabolize the yellow-colored soluble MTT into water-insoluble purple formazan crystals. After 4 h of incubation, the culture medium was removed, and the crystals were extracted with DMSO added in a volume of 200 µL. The plates were placed on a shaker for 10 min. Then, 150 µL of the solution was transferred onto a 96-well flat-bottom polypropylene plate. Absorbance at 550 nm was assessed using a microplate reader (ELx 800, Bio-Tek Instruments Inc., Winooski, VT, USA). MTT reduction was calculated as a percentage of the control group.

### 2.8. Cytokines Secretion Measurement

The measurement of interleukin 6(IL-6), soluble IL-6 receptor alpha (sIL-6Ralfa), sIL-6Rbeta, IL-8, IL-10, IL-11 IL-20, IL-32, and Pentraxin-3 (PTX3) was carried out 24 h after the application of PDT. To determine the level of cytokines, supernatants of cells on which PDT had a sublethal effect were selected, which allows the assessment of the immunomodulatory effect. Previous studies have used this procedure with OSCC and HGF-1 lines [20] and other cell lines [21,22]. Concentrations were assessed in supernatants using the Bio-Plex Pro^TM^ Assay and Bio-Plex^®^ 200 System (BIO-RAD Laboratories, Inc.). The multiplex assay method is commonly used to determine the concentration of cytokines in both clinical and preclinical studies [23,24]. All steps of the procedure were performed following the manufacturer’s instructions. The experiments were repeated 4 times (*n* = 4).

### 2.9. Statistical Analysis

The Shapiro–Wilk test was used to check the normality of distribution. In the case of the MTT test, normality of distribution was obtained, and the percentage of MTT reduction of the study group with the control group was compared using the Student’s t-test. Kruskal–Wallis analysis of variance (ANOVA) with post hoc analysis using the multiple comparison test was used for cytokine concentrations. The statistical analysis was performed in Statistica version 13 (TIBCO Software Inc., Palo Alto, CA, USA, 2017), and the charts were prepared using Excel^®^ (Microsoft 365, 2303 version). Values of *p* < 0.05 were considered statistically significant.

## 3. Results

### 3.1. Fluorescence Microscopy

Images of cells obtained in a fluorescence microscope with a fluorescein isothiocyanate filter showed no fluorescence for the control group of cells not treated with HY and increasing fluorescence after incubation with PS solutions for both cancer cells and fibroblasts. The results are consistent with those previously published [20].

### 3.2. MTT Cytotoxicity Assay

**Figure 1:**
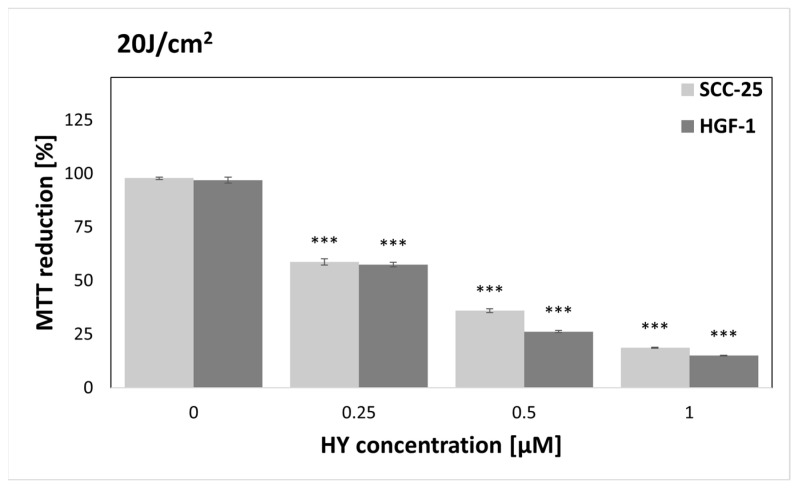
Percentage of MTT reduction by SCC-25 and HGF-1 cells after photodynamic therapy (PDT) using 0–1 µM hypericin (HY) doses and a 20 J/cm^2^ light dose. The values represent the means ± standard error (SE). *** *p* < 0.01.

MTT reduction was assessed for HY concentrations of 0, 0.25, 0.5, and 1 µM and light doses of 0, 1, 2, 5, 10, and 20 J/cm^2^ and compared to a control group of cells not treated with PS or light. In the dark, HY showed no cytotoxic effect on tumor cells. On the other hand, in the case of fibroblasts, HY with the highest concentration significantly reduced MTT reduction (MTT reduction = 82.88% ± 2.84%). In the case of the irradiation of cells with a dose of 1 J/cm^2^, a similar result was obtained in the form of HY cytotoxicity of 1 µM against HGF-1 cells (MTT reduction = 90.63% ± 5.06%). Interestingly, for the light dose of 2 J/cm^2^, no cytotoxic effect of PDT was demonstrated; however, for the HY concentration of 0.5 µM, a stimulating effect on fibroblasts was demonstrated (MTT reduction = 119.37% ± 10.16%). The repeated MTT study showed that the results were consistent with those previously published. Charts for doses of 0–10 J/cm^2^ are included in the Supplementary Material. Starting from a light dose of 5 J/cm^2^, PDT showed a cytotoxic effect on SCC-25 and HGF1 cells; the effect was stronger with increasing light dose and PS concentration (Figure 1). Therefore, supernatants of cells treated with 0 J/cm^2^, 1 J/cm^2^, and 2 J/cm^2^ and preincubated with doses of 0 µM, 0.25 µM, and 0.5 µM HY were used for further determinations.

### 3.3. Effect of HY-PDT on Secretory Activity: IL-6

**Figure 2:**
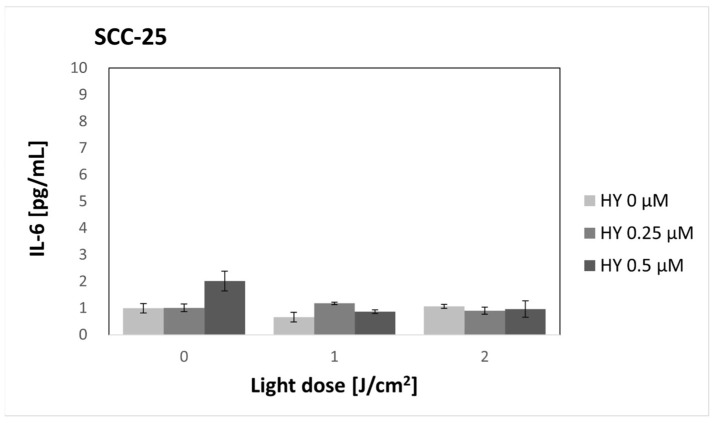
The concentration of interleukin 6 (IL-6) in the supernatants from the oral cancer cell culture SCC-25 line. The values represent the means ± SE.

**Figure 3:**
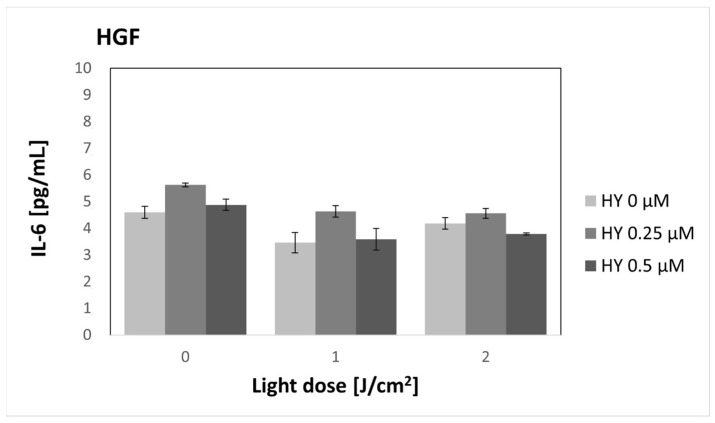
The concentration of IL-6 in the supernatants from the gingival fibroblast HGF-1 line. The values represent the means ± SE.

HY-PDT did not affect the secretion of IL-6 in both the SCC-25 and HGF-1 lines (Figure 2 and Figure 3). There was a significantly higher production of IL-6 by fibroblasts compared to cancer cells in the corresponding groups. In the case of cancer cells, the level of IL-6 for the dark control without PS was 1.00 pg/mL ± 0.18 pg/mL. For gingival fibroblasts in the corresponding group, it was 4.60 pg/mL ± 0.23 pg/mL.

### 3.4. Effect of HY-PDT on Secretory Activity: sIL-6Ralpha

**Figure 4:**
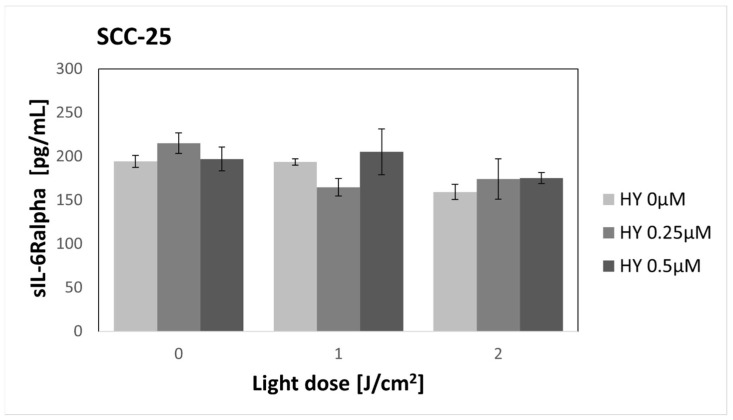
The concentration of sIL-6Ralpha in the supernatants from the oral cancer cell culture SCC-25 line. The values represent the means ± SE.

**Figure 5:**
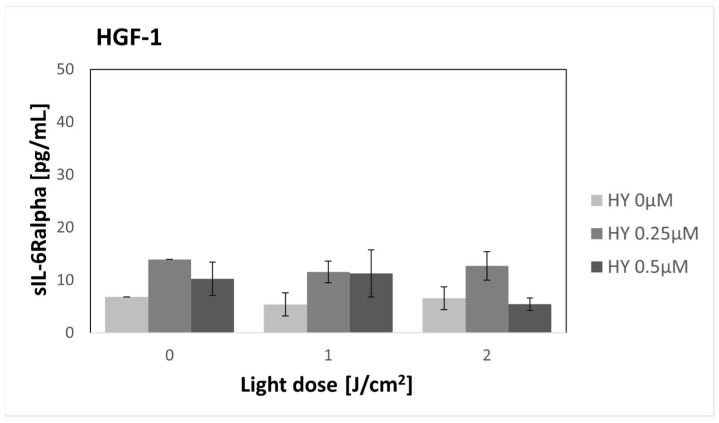
The concentration of sIL-6Ralpha in the supernatants from the gingival fibroblast HGF-1 line. The values represent the means ± SE.

There were no changes in the secretion of sIL-6Ralpha after the use of HY-PDT in both cancer cells and fibroblasts (Figure 4 and Figure 5). A significantly higher production of sIL-6Ralpha by cancer cells was found compared to fibroblasts in the corresponding groups. For the dark control without PS, in the case of SCC-25 cells, the sIL-6Ralpha level was 194.47 pg/mL ± 6.89 pg/mL, while for the HGF-1 line, it was 6.83 pg/mL.

### 3.5. Effect of HY-PDT on Secretory Activity: sIL-6Rbeta

**Figure 6:**
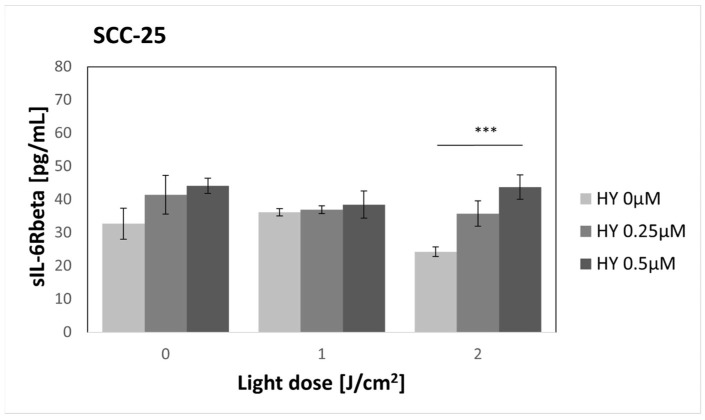
The concentration of sIL-6Rbeta in the supernatants from the oral cancer cell culture SCC-25 line. The values represent the means ± SE. The line over the bars indicates groups that were significantly different (Kruskal–Wallis Test and Dunn’s multiple comparisons tests). *** *p* < 0.01.

**Figure 7:**
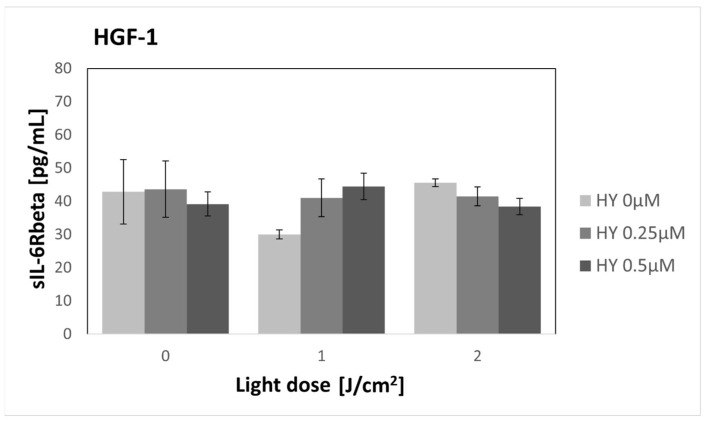
The concentration of sIL-6Rbeta in the supernatants from the gingival fibroblast HGF-1 line. The values represent the means ± SE.

HY alone did not cause significant changes in sIL-6Rbeta secretion (Figure 6). For the dark control without HY, sIL-6Rbeta production was found to be 32.69 pg/mL ± 4.69 pg/mL. In the case of cancer cells, a statistically significant change was found after applying a light dose of 2 J/cm^2^. The sIL-6Rbeta level for 0 µM HY was 24.25 pg/mL ± 1.46 pg/mL, and for 0.5 µM HY, it was 43.73 pg/mL ± 3.64 pg/mL. The level of sIL-6Rbeta did not change after PDT application on fibroblasts (Figure 7). For the dark control, a value of 42.87 pg/mL ± 9.69 was obtained.

### 3.6. Effect of HY-PDT on Secretory Activity: IL-8

**Figure 8:**
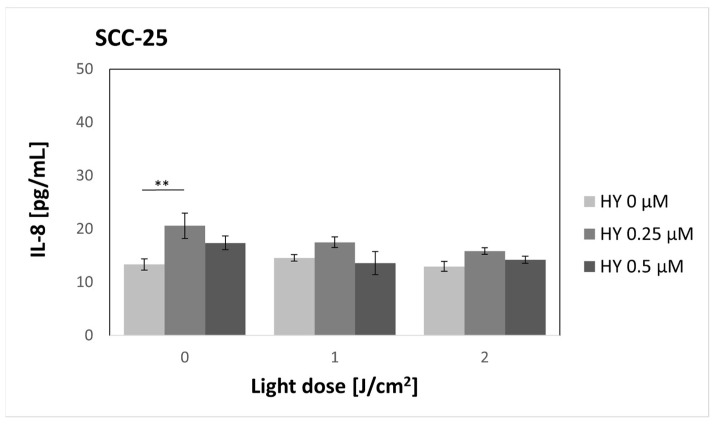
The concentration of IL-8 in the supernatants from the oral cancer cell culture SCC-25 line. The values represent the means ± SE. The line over the bars indicates groups that were significantly different (Kruskal–Wallis Test and Dunn’s multiple comparisons tests). ** *p* < 0.05.

There was an increase in the concentration of IL-8 for the SCC-25 line (Figure 8) between the control without PS and the 0.25 μM HY dose without irradiation. For the 0 µM dose, the cytokine concentration was 13.35 pg/mL ± 1.04 pg/mL, and for the 0.25 µM dose, it was 20.60 pg/mL ± 2.38 pg/mL. For other doses of the PS and light, no statistically significant differences were found in the case of cancer cells.

**Figure 9:**
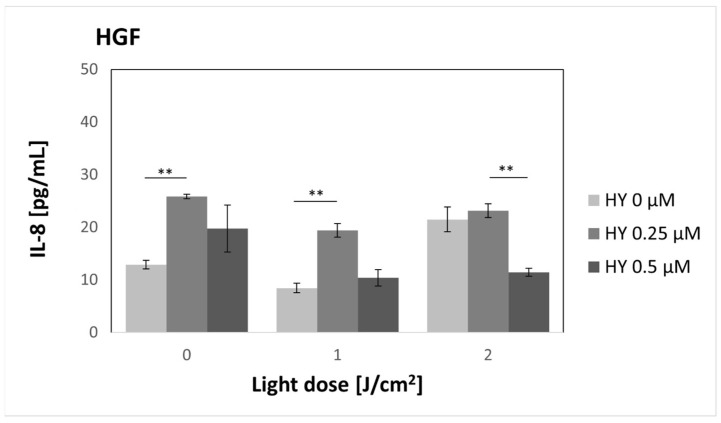
The concentration of IL-8 in the supernatants from the gingival fibroblast HGF-1 line. The values represent the means ± SE. The lines over the bars indicate groups that were significantly different (Kruskal–Wallis Test and Dunn’s multiple comparisons tests). ** *p* < 0.05.

As for the HGF-1 line (Figure 9), a statistically significant increase in IL-8 concentration was also demonstrated after using PS alone at a dose of 0.25 µM without the use of light compared to the dose of 0 µM HY; the concentration values were 25.85 pg/mL ± 0.42 pg/mL and 12.88 pg/mL ± 0.81 pg/mL, respectively. A similar picture is visible for the light dose of 1 J/cm^2^, where for the dose of 0 µM, the concentration of IL-8 was 8.46 pg/mL ± 0.89 pg/mL, and after PDT, with the use of 0.25 µM HY, it was 19.40 pg/mL ± 1.3 pg/mL. The situation is different at the light dose of 2 J/cm^2^, where after irradiation of cells not incubated with PS, there was an increase in the concentration of IL-8 (21.47 pg/mL ± 2.35 pg/mL). However, it was not a statistically significant increase compared to the dark control. After using HY, the 0.25 µM concentration remained similar (23.16 pg/mL ± 1.29). In the case of cells incubated with 0.5 µM HY, there was a significant decrease in IL-8 concentration (11.43 pg/mL ± 0.74).

### 3.7. Effect of HY-PDT on Secretory Activity: IL-10

**Figure 10:**
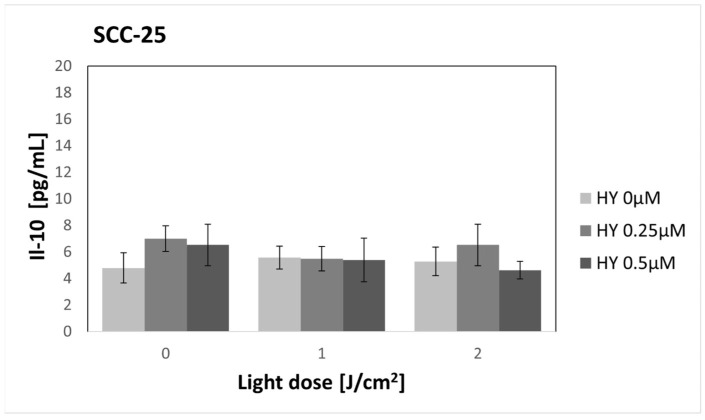
The concentration of IL-10 in the supernatants from the oral cancer cell culture SCC-25 line. The values represent the means ± SE.

**Figure 11:**
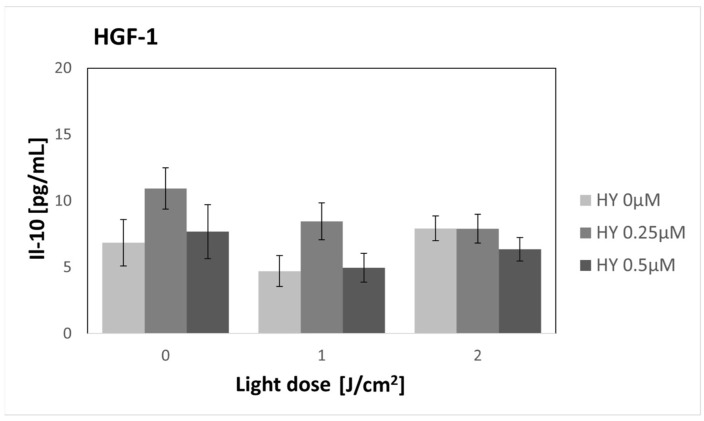
The concentration of IL-10 in the supernatants from the gingival fibroblast HGF-1 line. The values represent the means ± SE.

In the case of IL-10, no statistically significant differences were demonstrated for both the SCC-25 and HGF-1 cell lines (Figure 10 and Figure 11). In the case of SCC-25, the cytokine level was 4.80 pg/mL ± 1.14 pg/mL, while for HGF-1, it was 6.84 pg/mL ± 1.74 pg/mL.

### 3.8. Effect of HY-PDT on Secretory Activity: IL-11

**Figure 12:**
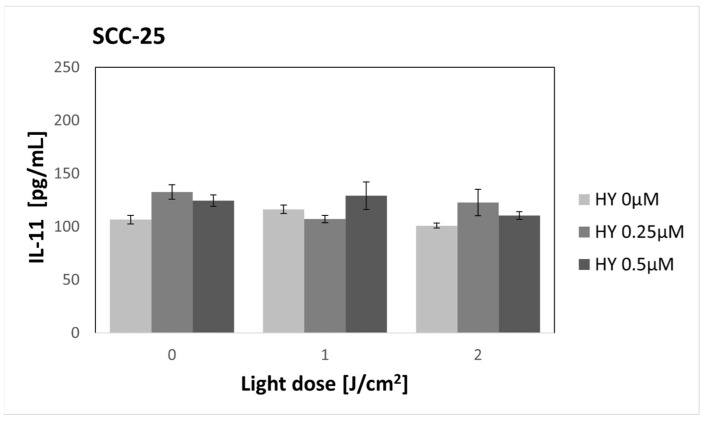
The concentration of IL-11 in the supernatants from the oral cancer cell culture SCC-25 line. The values represent the means ± SE.

**Figure 13:**
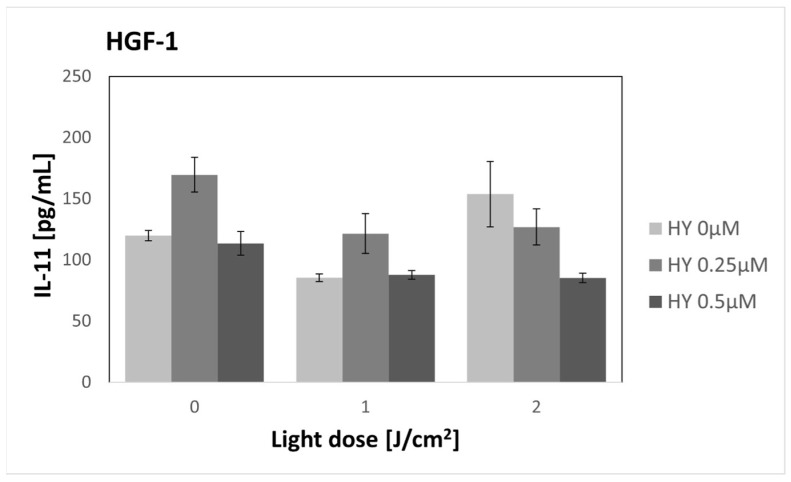
The concentration of IL-11 in the supernatants from the gingival fibroblast HGF-1 line. The values represent the means ± SE.

There were no changes in the concentration of IL-11 after using PDT in the case of the SCC-25 line (Figure 12). In the control group, the secretion reached 106.67 pg/mL ± 4.19. For HGF-1 (Figure 13), an increase in cytokine concentration was noted after using 0.25 µM HY alone (169.60 pg/mL ± 14.19 pg/mL) and 2 J/cm^2^ irradiation alone (153.75 pg/mL ± 26.66 pg/mL) compared to the control group (119.91 pg/mL ± 4.24 pg/mL). Those differences, however, were not statistically significant.

### 3.9. Effect of HY-PDT on Secretory Activity: IL-20

**Figure 14:**
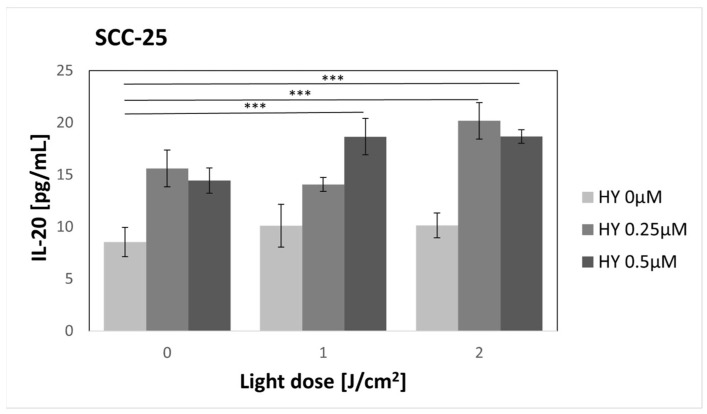
The concentration of IL-20 in the supernatants from the oral cancer cell culture SCC-25 line. The values represent the means ± SE. The lines over the bars indicate groups that were significantly different (Kruskal–Wallis Test and Dunn’s multiple comparisons tests). *** *p* < 0.01.

The use of HY in doses of 0.25 µM and 0.5 µM increased the secretion of IL-20 compared to the control group of SCC-25 (8.54 pg/mL ± 1.40 pg/mL) (Figure 14). However, it was not statistically significant. After using PDT, the secretion of IL-20 significantly increased for cancer cells incubated with 0.5 µM HY and irradiated with 1 J/cm^2^ (18.67 pg/mL ± 1.75 pg/mL). In the case of a light dose of 2 J/cm^2^, a statistically significant increase in the secretion of this cytokine was found for both 0.25 µM HY (20.18 pg/mL ± 1.73 pg/mL) and 0.5 µM HY (18.68 pg/mL ± 0.66 pg/mL). In the case of HGF-1 cells, no secretion of this cytokine was found.

### 3.10. Effect of HY-PDT on Secretory Activity: IL-32

**Figure 15:**
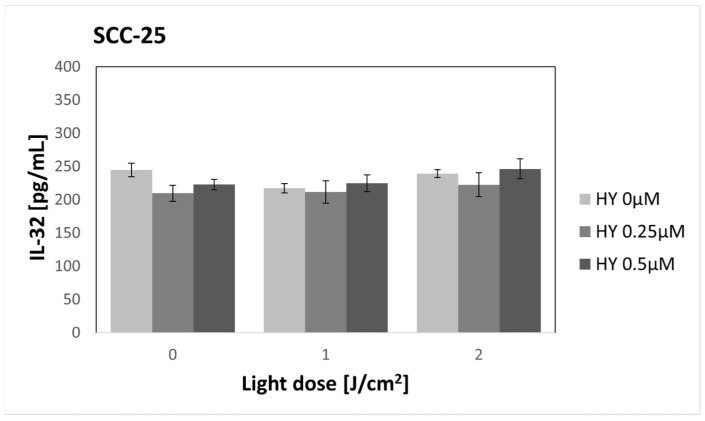
The concentration of IL-32 in the supernatants from the oral cancer cell culture SCC-25 line. The values represent the means ± SE.

**Figure 16:**
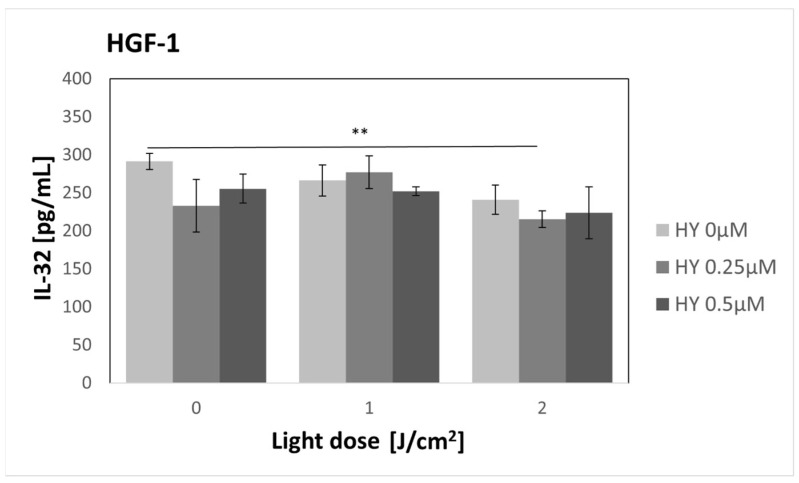
The concentration of IL-32 in the supernatants from the gingival fibroblast HGF-1 line. The values represent the means ± SE. The line over the bars indicates groups that were significantly different (Kruskal–Wallis Test and Dunn’s multiple comparisons tests). ** *p* < 0.05.

There was no change in IL-32 secretion after applying PDT to SCC-25 cells (Figure 15). In the control group, the concentration was 244.89 pg/mL ± 10.35 pg/mL. In the case of fibroblasts (Figure 16), there was a statistically significant decrease in the cytokine concentration after applying PDT at a dose of 2 J/cm^2^ and 0.25 µM HY compared to the control group. The values were 215.55 pg/mL ± 11.10 pg/mL and 291.51 pg/mL ± 10.63 pg/mL, respectively.

### 3.11. Effect of HY-PDT on Secretory Activity: PTX3

**Figure 17:**
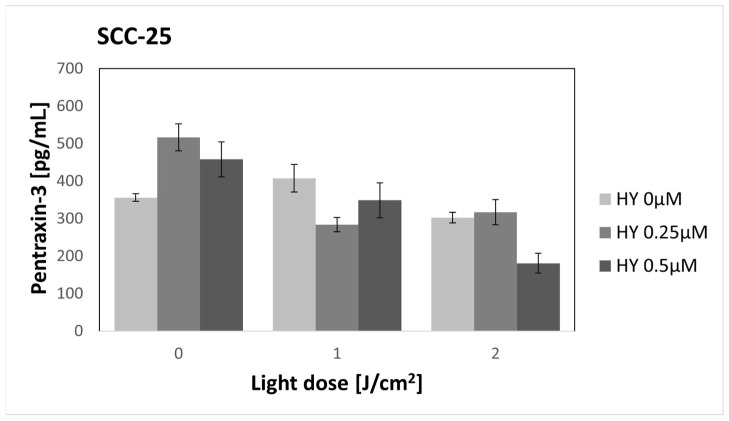
The concentration of PTX3 in the supernatants from the oral cancer cell culture SCC-25 line. The values represent the means ± SE.

The SCC-25 control group had a PTX3 secretion of 356.23 pg/mL ± 10.21 pg/mL. After HY alone, there was an insignificant increase in PTX3 secretion for both 0.25 μM HY (516.66 pg/mL ± 36.00 pg/mL) and 0.5 μM HY (458.04 pg/mL ± 46.78 pg/mL). After using HY-PDT, the PTX3 secretion decreased again. In the group with 0.5 μM HY and 2 J/cm^2^, the level was 180.91 pg/mL ± 26.36 pg/mL. However, there was no significant difference compared to the control group (*p* = 0.073) (Figure 17). There were statistical differences between the remaining groups but without any visible dependence on the dose of light or PS used. Therefore, this was not placed on the graph so as not to limit its readability. The level of the PTX3 secreted by the HGF-1 line was below the quantifiable value.

## 4. Discussion

The effect of HY-PDT on the secretion of selected cytokines by persistent OSCC cells was assessed. The tumor microenvironment (TME) of oral cancer is complex, consisting of cellular components and an extracellular matrix. Cellular components include immune cells and stromal cells. The main group of stromal cells are cancer-associated fibroblasts (CAF). Fibroblasts located in the TME may secrete cytokines, influencing tumor progression, metastasis, or escape from the immune system [25]. For this reason, the authors chose oral cancer cell lines (SCC-25) and fibroblasts (HGF-1) for this study.

Immune, healthy, and malignant cells are in a close relationship during cancer development. A cancer’s distinctive characteristic is its ability to combat the immune system. Cytokines play a key role in intercellular interactions in a tumor environment. They have concentration-dependent and multidirectional effects due to the multitude of cells that secrete them, the presence of diverse receptors, and signaling pathways. Therefore, the cytokine can be responsible for the initiation, progression, and, even further, the inhibition of tumor development [26].

Cancer cells can be characterized by overexpression of certain cytokines groups, e.g., IL-6 or IL-11. These act in an autocrine manner, leading to increased proliferation, stimulation of migration, inhibition of apoptosis, and further production of cytokines, e.g., IL-8. IL-8, among others, that is secreted by the tumor may promote an immunosuppressive tumor environment by recruiting polymorphonuclear leukocytes [27]. Il-6 and Il-8 are considered oncogenic cytokines because they are associated with epithelial-mesenchymal transition, disrupting intercellular interactions, hindering macrophage function, and promoting cancer cell invasion. Increased levels of these cytokines were found in patients with OSCC [28].

Unlike many soluble interleukin receptors, which act antagonistically when bound to interleukin, the Il-6/sIl-6R complex acts agonistically and will broaden the spectrum of the cells it acts on via membrane-bound gp130. This complex influences cell proliferation, differentiation, and regulation of plan-state mediators and also extends the half-life of Il-6. IL-11 also signals through gp130 and therefore has an overlapping effect with the mentioned complex and may mimic the stimulatory properties of Il-6/sIl-6R [29,30].

In the tumor microenvironment, IL-10 has a pleiotropic effect. It can both activate cytotoxic T lymphocytes and have an immunosuppressive effect, inhibiting the secretion of IL-12 and the inflammatory process [27]. Immunosuppressive cytokines may have a bidirectional effect, inhibiting the pro-inflammatory effects leading to tumor progression, or may reduce the anti-tumor immune response. Also, the concentration of IL-10 may be increased in patients with OSCC, and its increased expression is associated with a more aggressive type of OSCC [28]. IL-20 has a tumor growth-promoting effect by inducing programmed cell death protein [27].

Fibroblasts participate in the immune response that involves the secretion of pro-inflammatory cytokines and the presentation of antigens to T lymphocytes. The secretion of cytokines by fibroblasts depends on the stimulating factor. It is controlled at the level of transcription, translation, and post-translational processing, among others [31]. CAFs play a key role in cancer development. They can induce tumor immunosuppression through the secretion of, for instance, IL-6 and IL-32 [32,33].

Determining cytokine secretion after PDT can allow for a better understanding of the mechanisms of the therapy’s immunomodulatory effect.

The use of PDT in the treatment of pre-cancerous lesions as well as OSCC is extensively studied in in vitro preclinical studies [17], an animal model [16], and clinical trials [34]. The results of these studies encourage further study of the mechanisms of PDT action and the search for the most effective procedure for a given type of cancer. In addition to the direct cytotoxic effect, an important mechanism is an immunomodulatory effect [1]. Our study used HY as a PS and an incoherent light source for photodynamic action. Such a procedure has been reported previously in studies on colorectal cancer cells [35] and our earlier OSCC studies [20]. Using sublethal doses, we demonstrated the immunomodulatory effect of PDT and changes in the secretion of cytokines such as sIL-6Rbeta, IL-8, IL-20, IL-32, and PTX3.

According to the authors’ best knowledge, this is the first study using Hypericin in PDT of OSCC and assessing its effect on the secretion of the cytokines mentioned above by cancer cells and fibroblasts potentially located in the TME. The cytokines were selected based on their previously described effects on cancer development and their interactions.

In our study, we repeated the MTT cytotoxicity test, and the results were consistent with those obtained in the previous study. Due to the reproducibility of HY-PDT cytotoxicity results, we refer readers to the Supplemental Material and the authors’ earlier studies for detailed results [20]. In our study, we found no cytotoxic effect of HY in the dark, except for the highest dose of PS applied to fibroblasts. A similar effect was found for a light dose of 1 J/cm^2^. The cytotoxic effect of HY has already been observed in the dark at higher concentrations [36]. In the case of light irradiation of 2 J/cm^2^, a stimulating effect on HGF-1 cells was found when 0.5 μM HY was used; moreover, no phototoxic effect of 1 μM HY was observed as in the case of lower light doses. Authors suspect that this result is related to the photobiomodulating effect of subthreshold doses of light. Etemadi et al. demonstrated the stimulating effect of low light doses on healthy gingival fibroblasts [37]. Starting from 5 J/cm^2^, we found increasing cytotoxicity of PDT against both the SCC-25 and HGF-1 lines, with increasing light and PS doses. A linear increase in HY cytotoxicity with increasing concentration towards HNSCC cells was also observed by Bublik et al. [38].

The cytotoxic effect was observed both against cancer cells and healthy gingival fibroblasts, which indicates the lack of selectivity of HY towards cancer cells. This also indicates the need to target the cancer cells to avoid damage to healthy tissues. Similar observations were found in a study of leukemic and healthy bone marrow cells [39] In the case of studies on 2D monocultures, both cancer and healthy cells have direct contact with PS dissolved in the culture medium; therefore, HY easily enters both groups of cells [40].

It is widely known that cytokines are secreted by immune system cells; they play a vital role in both inhibition and progression of the cancer process. However, in addition to the immune cells, cancer cells themselves can produce cytokines [27]. Only living cells can actively secrete proteins; therefore, to determine the influence of cancer cells on the immunomodulatory effect of PDT, tests were performed for sublethal doses. Moreover, from a clinical point of view, it is important to understand the behavior of residual cancer cells in the event that the treatment is not radical, as well as the response of accompanying healthy cells.

Our previous studies on HY-PDT showed increased secretion of the soluble receptor for tumor necrosis factor alpha by SCC-25 cells after applying sublethal doses of light and PS [20]. Kaleta-Richter et al. showed the effect of sublethal doses of HY-PDT on the secretion of IL-8 depending on the malignancy of the cells. However, they did not show the effect of HY-PDT on the secretion of IL-10 [35]. These results correlate with our results because we also, in our experiments, did not obtain statistically significant differences after using PDT on IL-10 secretion. In the case of IL-8, in our experiment, we noted the stimulating effect of HY alone without light in both the HGF-1 and SCC-25 lines. No such effect was found in the study on colorectal cancer cells. Similar results were obtained by Du et al. on nasopharyngeal cells. They also found an increase in IL-8 production after using HY alone. At the same time, HY-PDT did not affect the production of this cytokine. They did not find any effect of HY and HY-PDT on the level of IL-10 [41].

Barathan et al. used HY-PDT against a human hepatocellular liver carcinoma cell line and found an increase in IL-6 secretion after the therapy. The most significant increase was noted when the cells were preincubated with HY at a concentration of 1.98 μM. Similarly, they found an increase in the secretion of IL-4, IL-10, and IFN-gamma, but at much lower values [42]. Our studies did not find statistically significant differences in the secretion of IL-6 and IL-10. However, the highest dose for which we determined the secretion of cytokines was 0.5 μM HY. In in vitro and in vivo studies on nasopharyngeal carcinoma cells, Du et al. found that the level of IL-6 gene expression and IL-6 secretion after applying HY-PDT depends on the degree of histological differentiation, basal cytokine production, and cell type. Researchers found higher levels of IL-6 in less differentiated cells [43].

IL-6 has two different signaling pathways. It can bind to a membrane receptor (mIL-6R) or a soluble (sIL-6R). The classical pathway is restricted to cells expressing a membrane-bound receptor. Proteolytic cleavage generates sIL-6R, which also can bind IL-6. The IL-6/sIL-6R complex can stimulate cells that do not have a receptor on their surface in the mechanism of the so-called trans-signaling [44]. We have demonstrated the production of sIL-6Ralpha and sIL-6Rbeta by the SCC-25 cells and that using PDT can affect the level of sIL-6Rbeta. Hwang et al. showed that sIL-6R is involved in osteoclast formation stimulated by OSCC [45]. Wang et al., examining samples from patients with OSCC, found a significantly higher expression of IL-6R and IL-6 mRNA transcripts in tumor tissue compared to healthy tissue. Receptor overexpression was associated with more histologically advanced and more extensive tumors [46].

We found increased IL-20 secretion by the SCC-25 cells after applying HY-PDT. IL-20 belongs to the IL-10 family and is a pro-inflammatory cytokine that plays an essential role in developing inflammatory diseases, such as psoriasis and rheumatoid arthritis [47]. Hsu et al. showed a higher expression of IL-20 and its receptors in the tumor tissue of patients with OSCC. The level correlated with the advancement of the tumor, which may indicate the involvement of this cytokine in the pathogenesis of OSCC. In in vitro studies, they found that IL-20 caused increased tumor cell proliferation and ROS production. In turn, administering an IL-20 inhibitor reduced proliferation and ROS production. Incubation of the OSCC cells with IL-20 increased expression of the pro-inflammatory cytokines TNF-α, IL-1β, and MCP-1, but not IL-6, IL-8, matrix metalloproteinase-2 (MMP-2), MMP-7, and MMP-9. In addition, IL-20 promoted tumor cell colony formation on agar, and a monoclonal antibody to IL-20 inhibited tumor growth in vivo. A possible cause of increased IL-20 secretion by cells is hypoxia [48]. Chen et al. found an increase in the production of IL-20 by HaCaT cells, keratinocytes, renal epithelial cells, monocytes, and chondrocytes in a hypoxic environment and increased levels of the cytokine after experimental ischemic stroke in a rat [49].

The function of IL-32 is not fully understood in cancer development. Studies indicate that it affects proliferation and metastasis by affecting the NF-κB pathway, STAT3, and MAPK signaling [50]. Guenina et al., in a study of OSCC patients, found that IL-32 overexpression was associated with reduced patient survival due to a potential role in metastasis [51]. Our study found that HY-PDT did not significantly affect IL-32 production by SCC-25 but decreased IL-32 production by HGF-1 cells. Wen et al. showed in their study on cancer-associated fibroblast (CAF) that IL-32-secreting fibroblasts promoted invasion and metastasis of breast cancer. They found that IL-32, interacting with β3 integrin, plays a significant role in the invasiveness of breast cancer. CAF-derived IL-32 bound to β3 integrin activating the MAPK pathway. This signaling resulted in the overexpression of fibronectin, N-cadherin, and vimentin. The knockdown of IL-32, β3 integrin, and blockade of MAPK signaling reduced the invasiveness of breast cancer [33].

PTX3 plays a key role in innate immunity. It can be overexpressed in many types of cancer and act as a prognostic factor. Its diverse activity in oncogenesis can play anti-cancer and pro-cancer roles [52]. Chang et al. found that malignant and metastatic HNSCC cells overexpress the PTX3 gene. EGF-induced PTX3 expression followed the PI3K/Akt and NF-κB pathways. Autocrine secretion of PTX3 increased the production of fibronectin and MMP-9, which promoted metastasis [53]. Chan et al. found that oleate increased PTX3 expression and secretion by activating the Akt/NF-κB pathway in HNSCC cells. Depleting PTX3 and inhibiting NF-κB reduced migration and invasiveness of HNSCC cells. PTX3 depletion also reduced the oleate-induced epithelial-mesenchymal transition markers vimentin and MMP-3. In addition, after PTX3 depletion, the researchers found that lung metastasis was blocked in mice [54]. In a study of cervical cancer, Ying et al. found that increased PTX3 expression was associated with tumor progression, and PTX3 knockdown resulted in reduced tumor cell viability, impaired colony formation, cell cycle arrest in the G2/M phase, and inhibition of MMP-2, MMP-9, and urokinase plasminogen activator. Blockade of PTX3 resulted in reduced carcinogenicity in mice and potential for lung metastasis [55].

There is evidence that HY can act on cells independently without irradiation. Our study demonstrated the cytotoxic effect of HY on HGF-1 cells at a concentration of 1 µM, while Besic Gyenge et al. demonstrated the cytotoxic effect of HY also on HSCC cells in the dark [36]. Huntosova et al. studied the effect of light-independent HY on glioma cells and benign endothelial cells. It showed faster uptake of HY by malignant cells, which was explained by the likely faster proliferation and metabolism of malignant cells. HY caused hyperpolarization of the mitochondrion and increased reactive oxygen species (ROS), but this was at a lower level than that induced by PDT. Moreover, HY caused an increase in the share of glycolysis in the cellular metabolism of malignant cells, indicating the PS’s ability to slow down the metabolism. HY in the dark showed a cytotoxic effect, and a higher level of toxicity was noted for endothelial cells than for glioblastoma [56]. Martínez-Poveda et al. showed on endothelial cells that HY not activated by light had an IC50 of 10 µM, while it had an IC50 of 13 nM when activated by light. In addition, HY in the dark inhibited endothelial tube formation on Matrigel, reduced urokinase production, and had an inhibitory effect on the wound healing assay [57].

HY exhibits absorption of electromagnetic radiation with a wavelength of 500 to 620 with a maximum at 595 nm [58]. Light of the mentioned length is characterized by poor penetration into tissues; therefore, in clinical conditions, it would enable the therapy of superficial precancerous lesions and the initial stages of cancer development [59]. The PS used is characterized by poor solubility in water, which requires modification with other ingredients before use [58].

A limitation of our experiment is that the study was conducted on a monolayer of cells. Such a model does not reflect the actual structure of the tumor or tissues. There is no extracellular matrix and proper intercellular interaction. The above-mentioned factors can affect cell proliferation, viability, and secretory activity, including cytokine secretion. In addition, the cultured cells have unlimited access to nutrients and oxygen, but also to the ingredients being tested [40,60]. In fact, the tumor structure is complex and there are other cells in the tumor environment, so what is important in determining the immunomodulatory effect of therapy is to see whether there are cells of the immune system. Among the immunomodulatory effects are those on the tumor microenvironment and intercellular interactions, where both cancer cells and immune cells coexist. Therefore, confirming the research results achieved with monolayer-cultured cells in spheroid studies with co-culture of immune cells, animal studies, and preferably in clinical trials is vital.

## 5. Conclusions

We confirmed the cytotoxic effect of HY-PDT on oral cancer cells and fibroblasts. We showed the immunomodulatory effect of sublethal doses of PDT and the possible effect of HY alone without irradiation. HY-PDT affected the SCC-25 cell secretion of sIL-6Rbeta, IL-20. HY alone increased the IL-8 secretion. In the case of HGF-1, the effect of HY-PDT on the secretion of IL-8 and IL-32 was found. There were no statistically significant differences in the secretion of IL-6, sIL-6Ralpha, IL-10, IL-11, or Pentraxin-3. However, no dependence on the change in the secretion of the above-mentioned cytokines on the increase in the dose of light or hypericin was observed.

## Data Availability

The data can be shared up on request.

## Supplementary Materials

The following supporting information can be downloaded at: https://www.mdpi.com/article/10.3390/pharmaceutics16010042/s1, Figure S1: Images of SCC-25 cells taken using an inverted fluorescence microscope. (A) Control group without PS. (B) PS absorption after incubation with 0.25 μM HY. (C) PS absorption after incubation with 0.5 μM HY. (D) PS absorption after incubation with 1 μM HY; Figure S2: Images of HGF-1 cells taken using an inverted fluorescence microscope. (A) Control group without PS. (B) PS absorption after incubation with 0.25 μM HY. (C) PS absorption after incubation with 0.5 μM HY. (D) PS absorption after incubation with 1 μM HY; Figure S3: Percentage of MTT reduction by SCC-25 and HGF-1 cells after PDT using 0–1 µM HY doses and a 0 J/cm^2^ light dose. The values represent the means ± SE. *** *p* < 0.01; Figure S4: Percentage of MTT reduction by SCC-25 and HGF-1 cells after PDT using 0–1 µM HY doses and a 1 J/cm^2^ light dose. The values represent the means ± SE. ** *p* < 0.05; Figure S5: Percentage of MTT reduction by SCC-25 and HGF-1 cells after PDT using 0–1 µM HY doses and a 2 J/cm^2^ light dose. The values represent the means ± SE. *** *p* < 0.01; Figure S6: Percentage of MTT reduction by SCC-25 and HGF-1 cells after PDT using 0–1 µM HY doses and a 5 J/cm^2^ light dose. The values represent the means ± SE. ** *p* < 0.05, *** *p* < 0.01; Figure S7: Percentage of MTT reduction by SCC-25 and HGF-1 cells after PDT using 0–1 µM HY doses and a 10 J/cm^2^ light dose. The values represent the means ± SE. *** *p* < 0.01.
