# Supplementary figures and images for: Immunomodulatory Effect of Hypericin-Mediated Photodynamic Therapy on Oral Cancer Cells
Source: Pharmaceutics. 2023 Dec 27;16(1):42. doi: 10.3390/pharmaceutics16010042 (PMC10819034; doi:10.3390/pharmaceutics16010042)

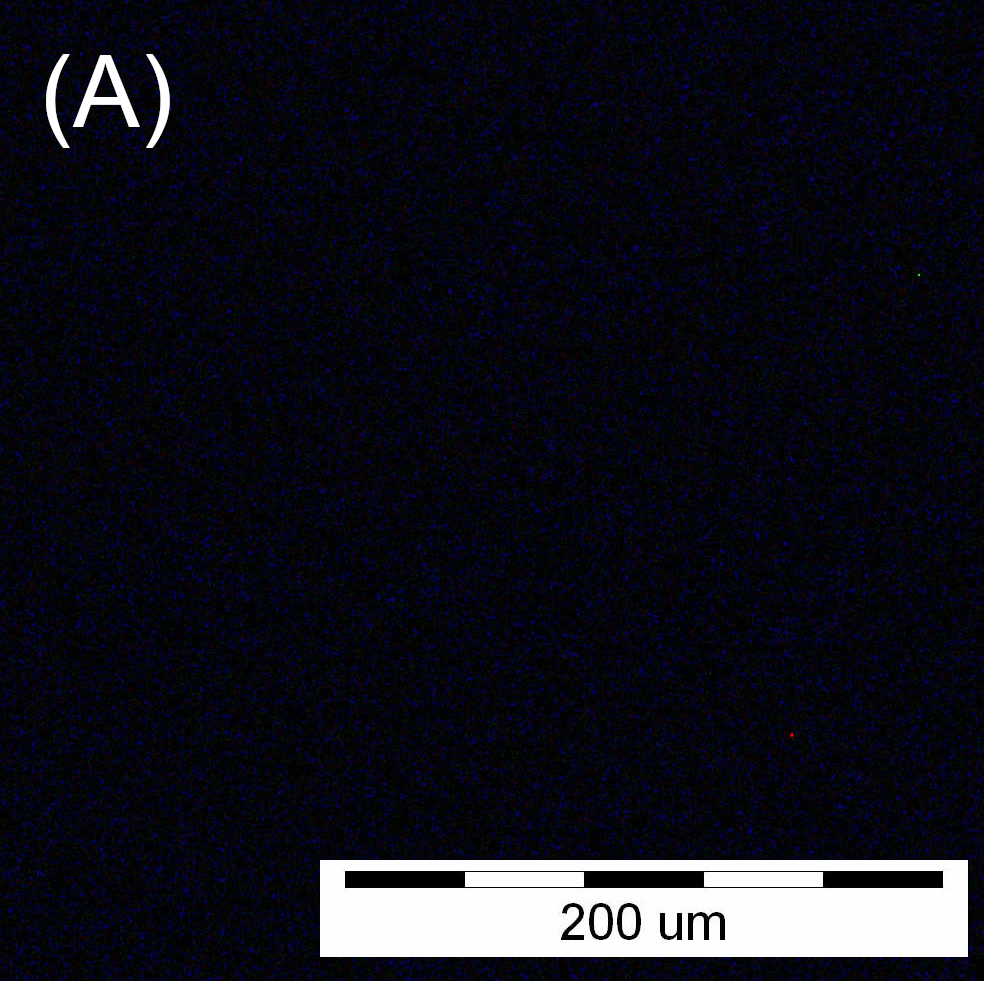

Supplement: Supplementary file 1 [file pharmaceutics-16-00042-s001.zip › Figure S1A.tiff]

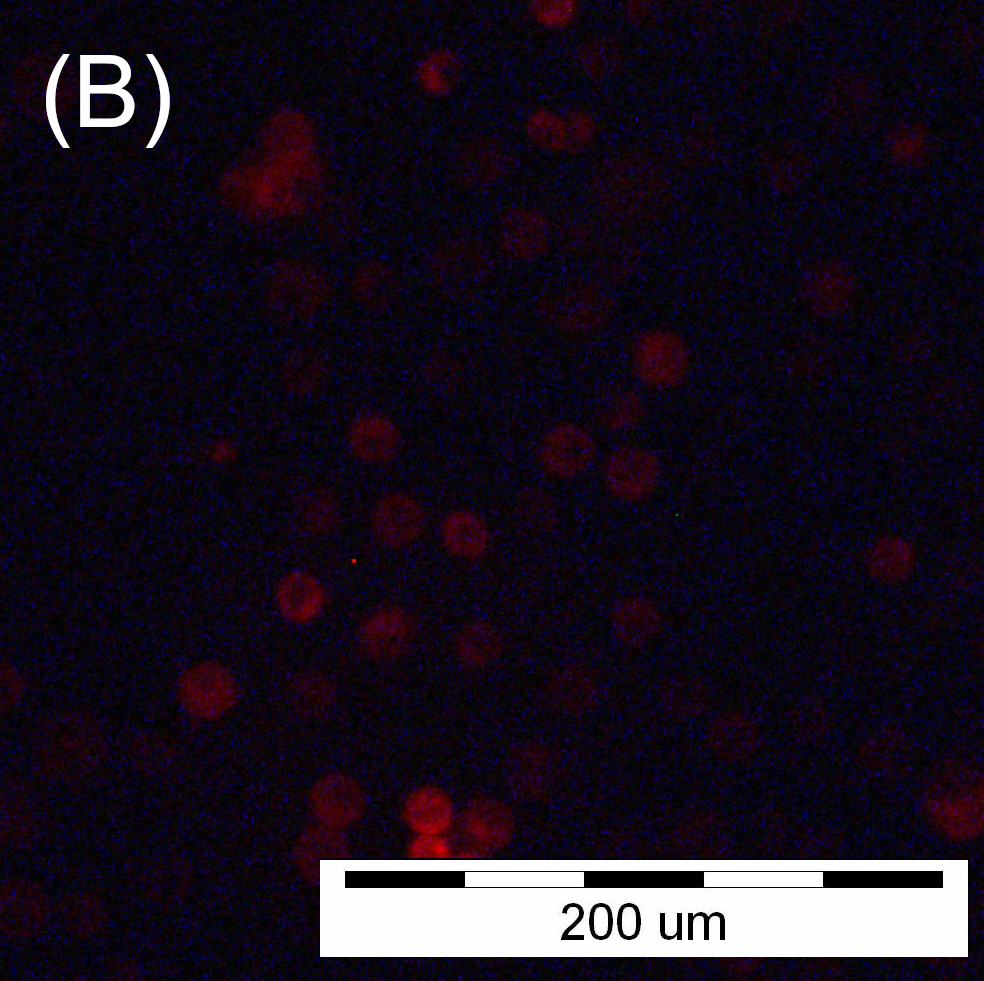

Supplement: Supplementary file 1 [file pharmaceutics-16-00042-s001.zip › Figure S1B.tiff]

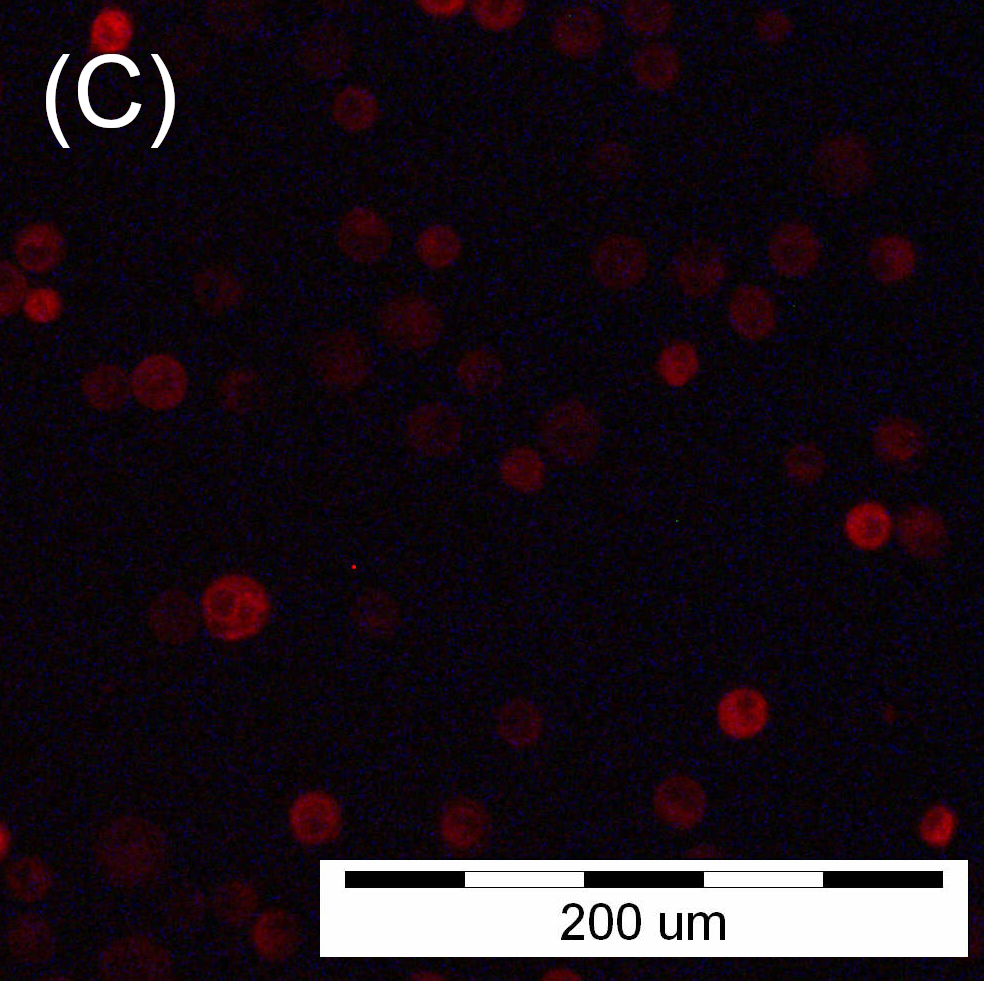

Supplement: Supplementary file 1 [file pharmaceutics-16-00042-s001.zip › Figure S1C.tiff]

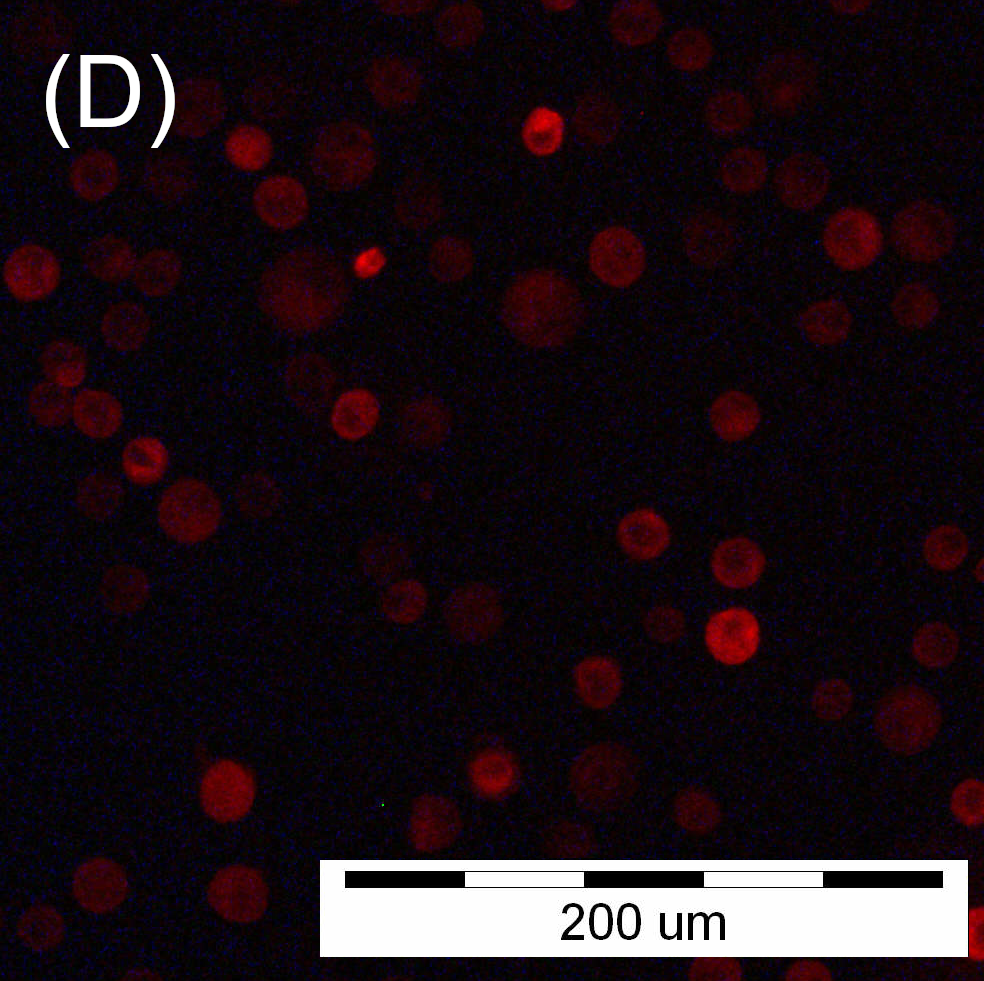

Supplement: Supplementary file 1 [file pharmaceutics-16-00042-s001.zip › Figure S1D.tiff]

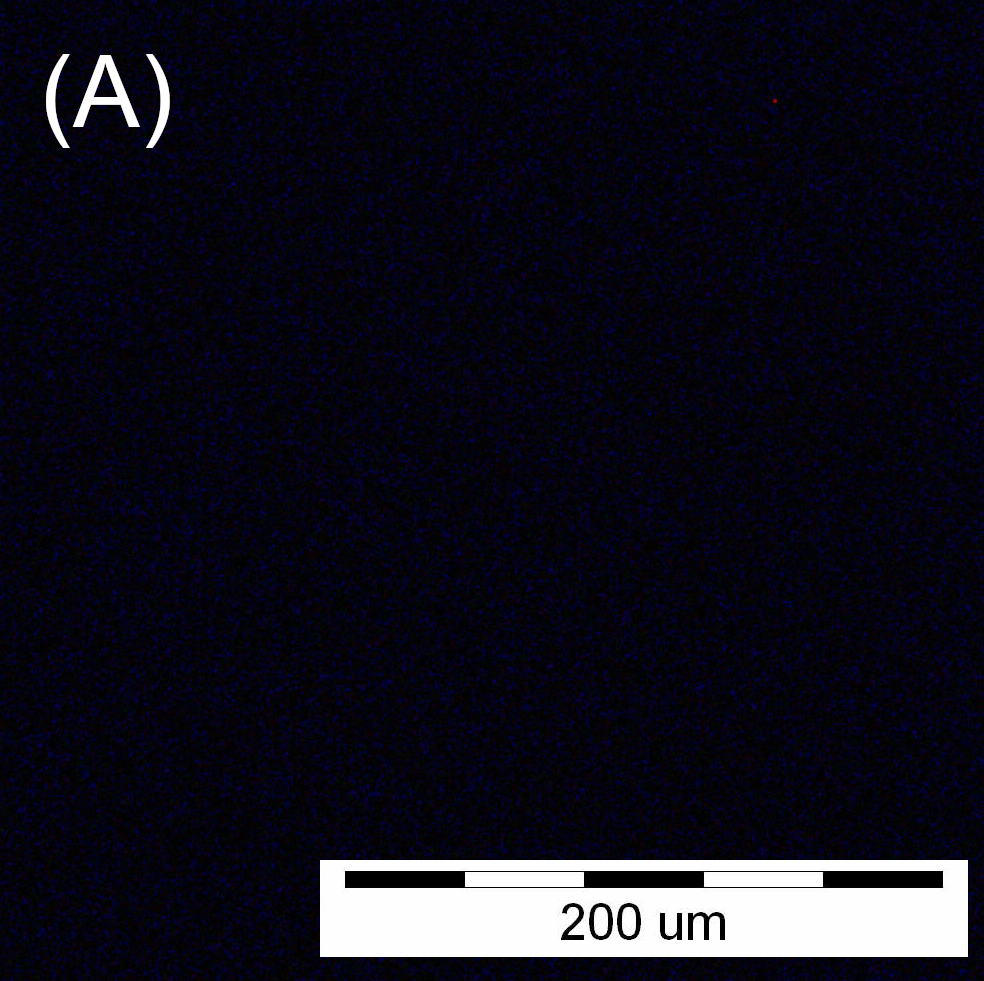

Supplement: Supplementary file 1 [file pharmaceutics-16-00042-s001.zip › Figure S2A.tiff]

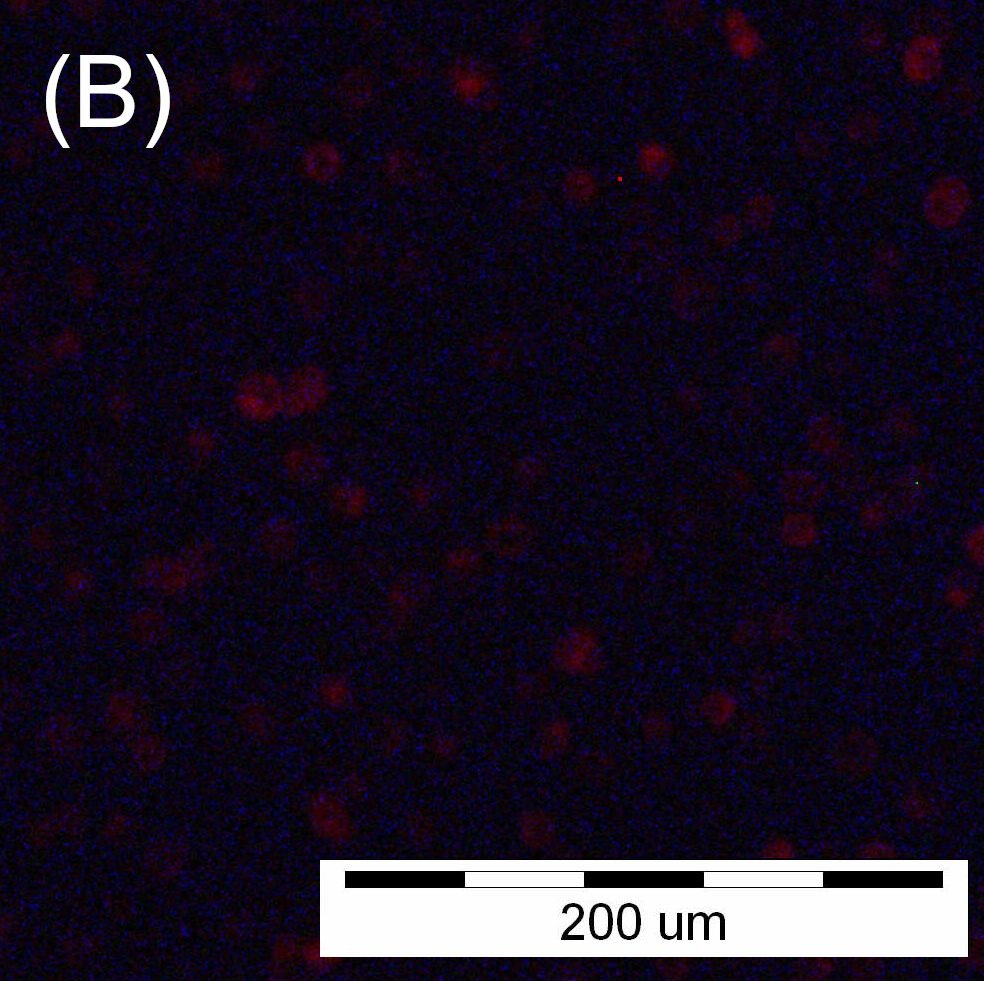

Supplement: Supplementary file 1 [file pharmaceutics-16-00042-s001.zip › Figure S2B.tiff]

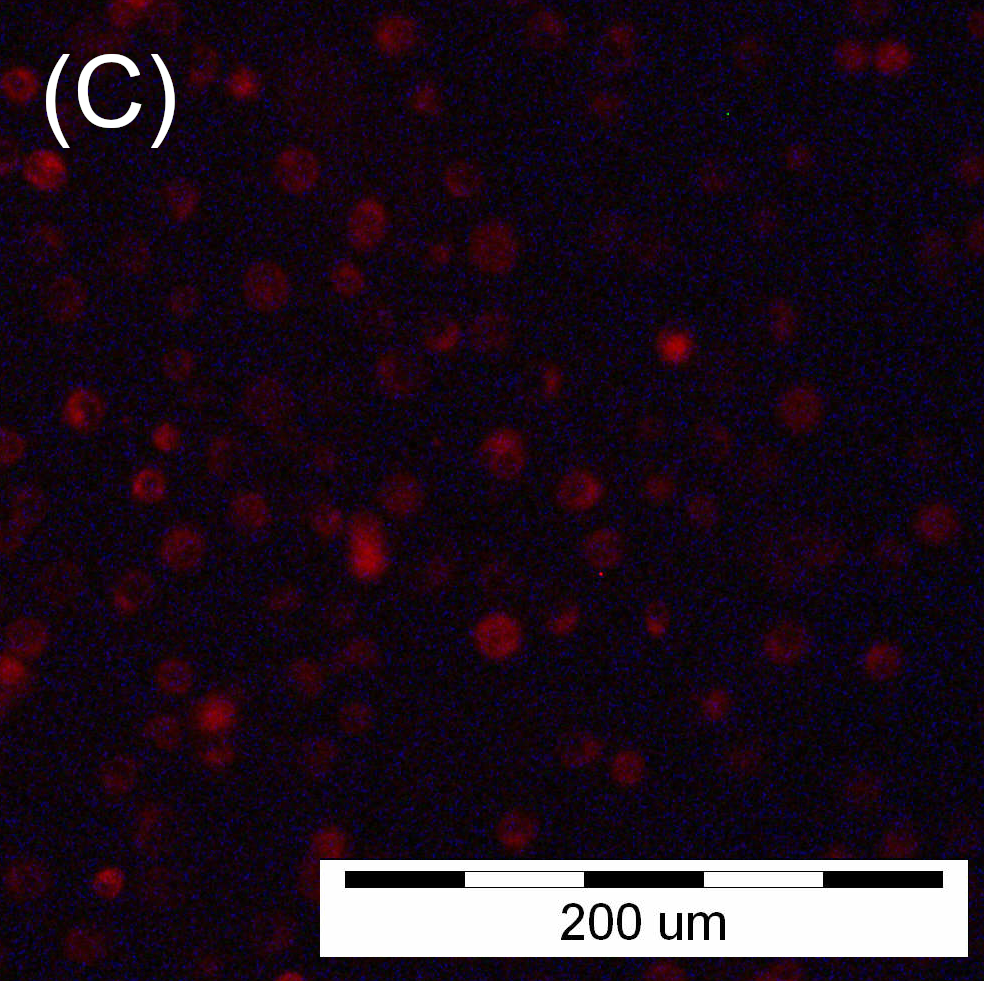

Supplement: Supplementary file 1 [file pharmaceutics-16-00042-s001.zip › Figure S2C.tiff]

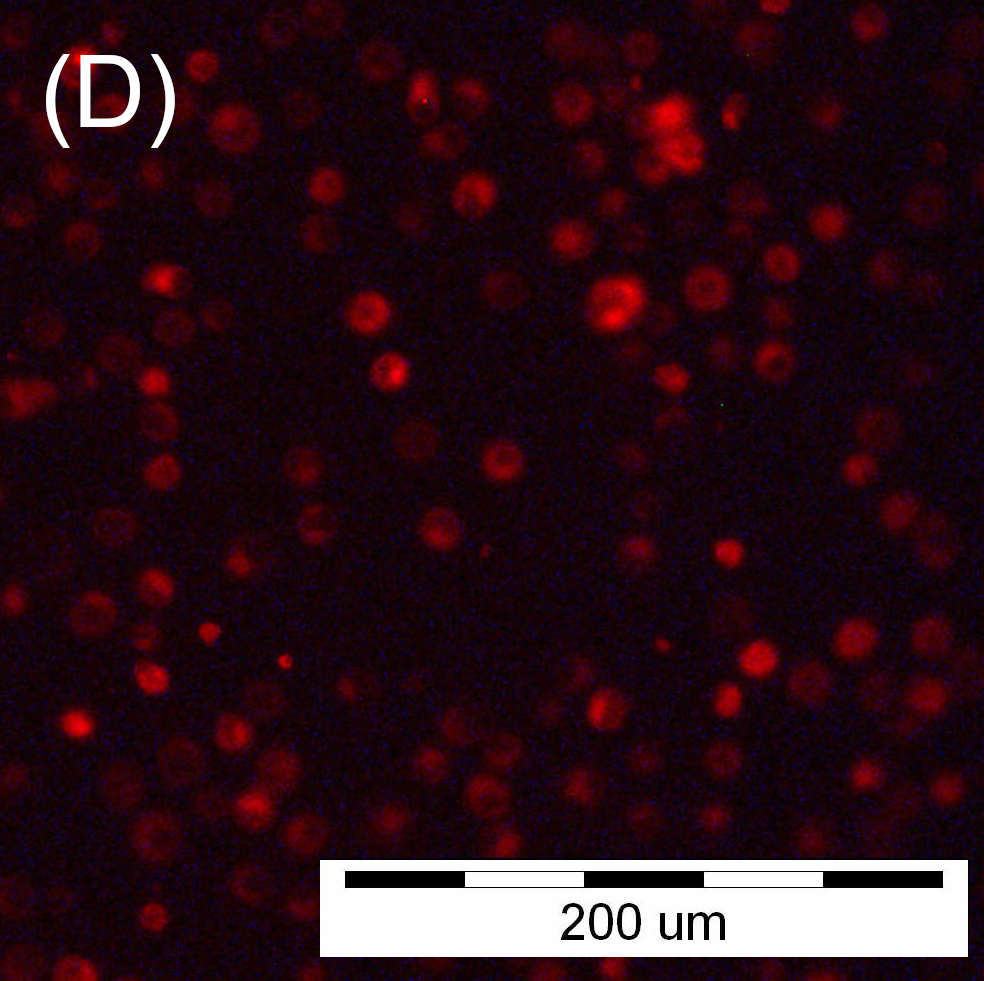

Supplement: Supplementary file 1 [file pharmaceutics-16-00042-s001.zip › Figure S2D.tiff]

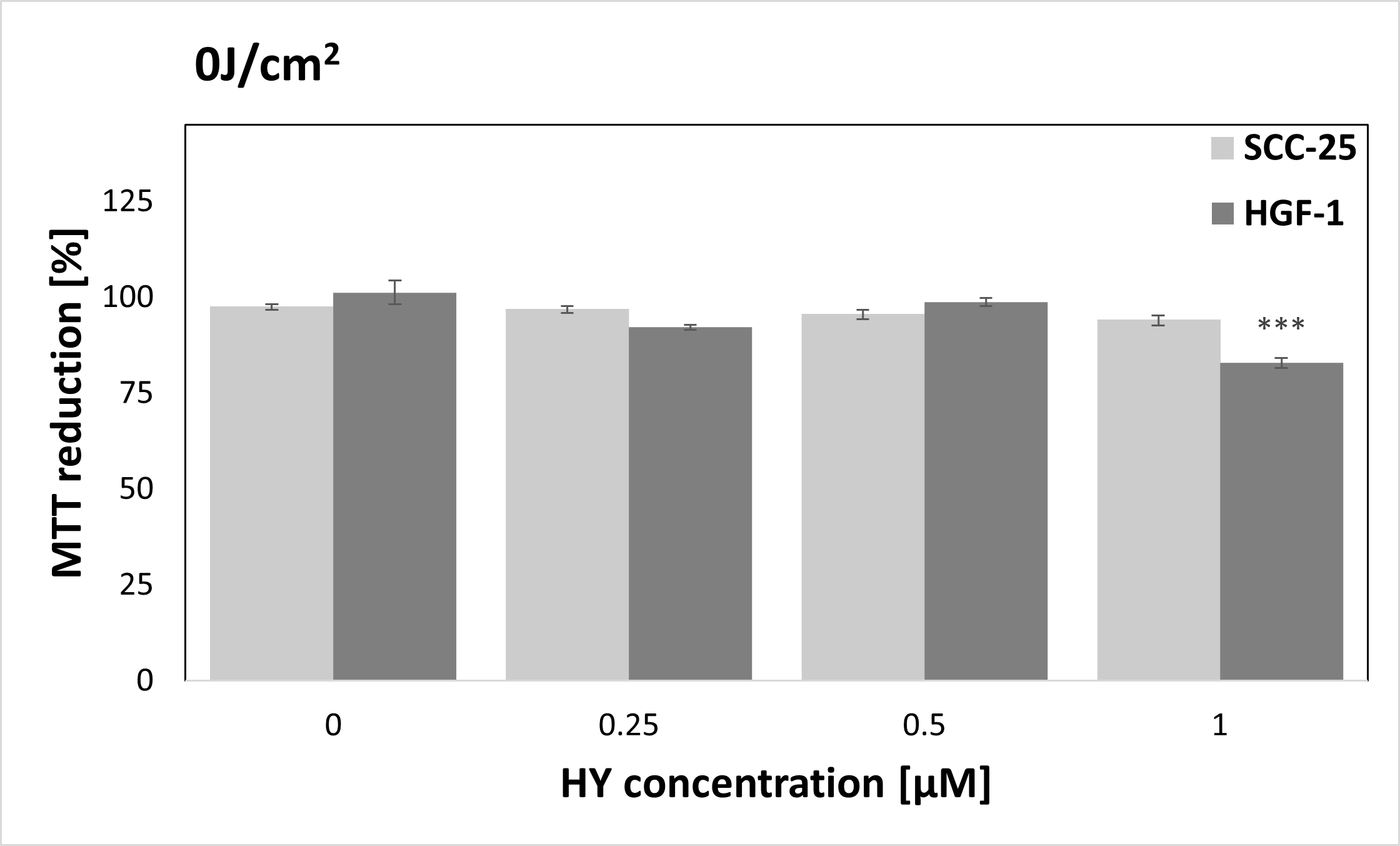

Supplement: Supplementary file 1 [file pharmaceutics-16-00042-s001.zip › Figure S3.png]

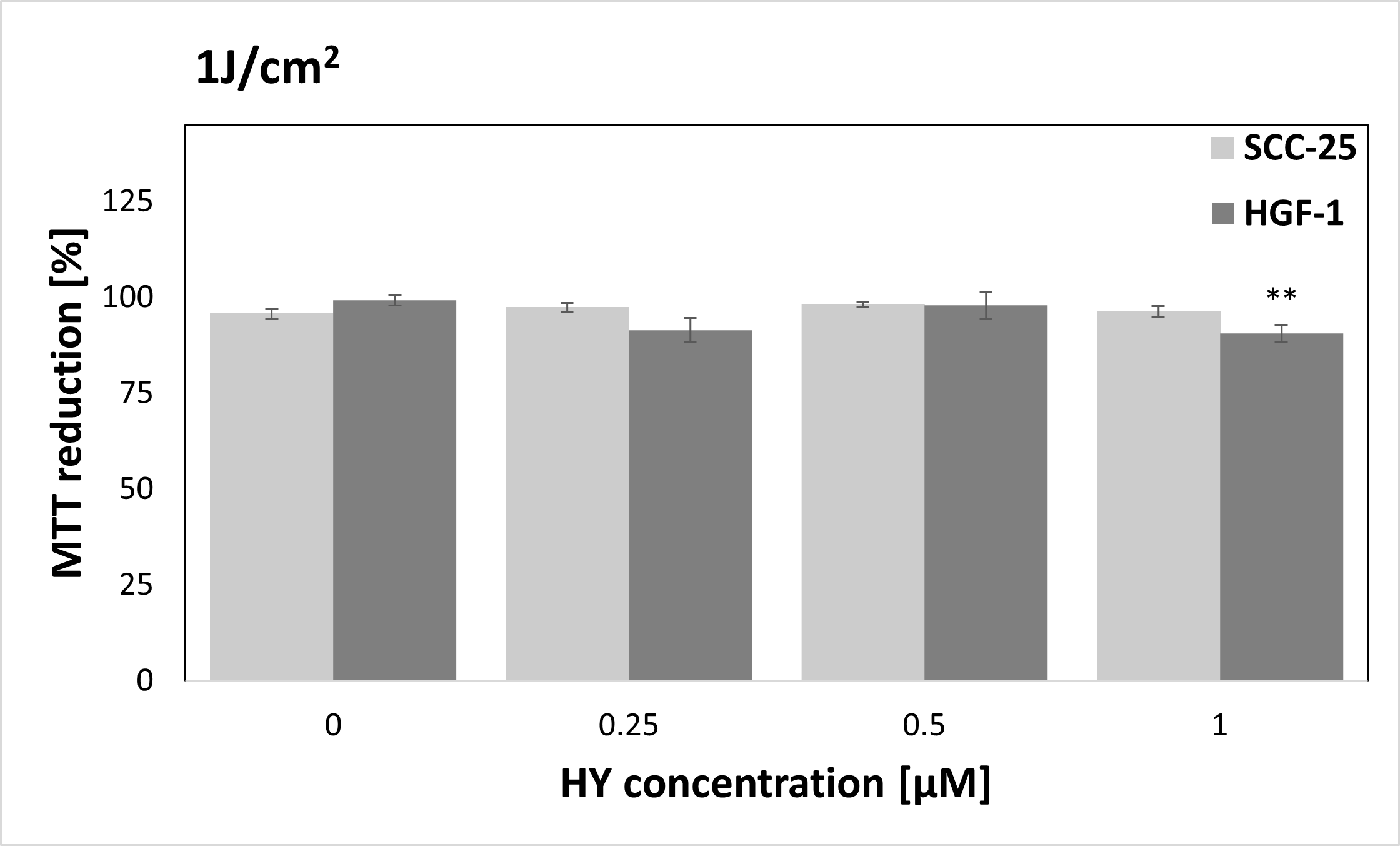

Supplement: Supplementary file 1 [file pharmaceutics-16-00042-s001.zip › Figure S4.png]

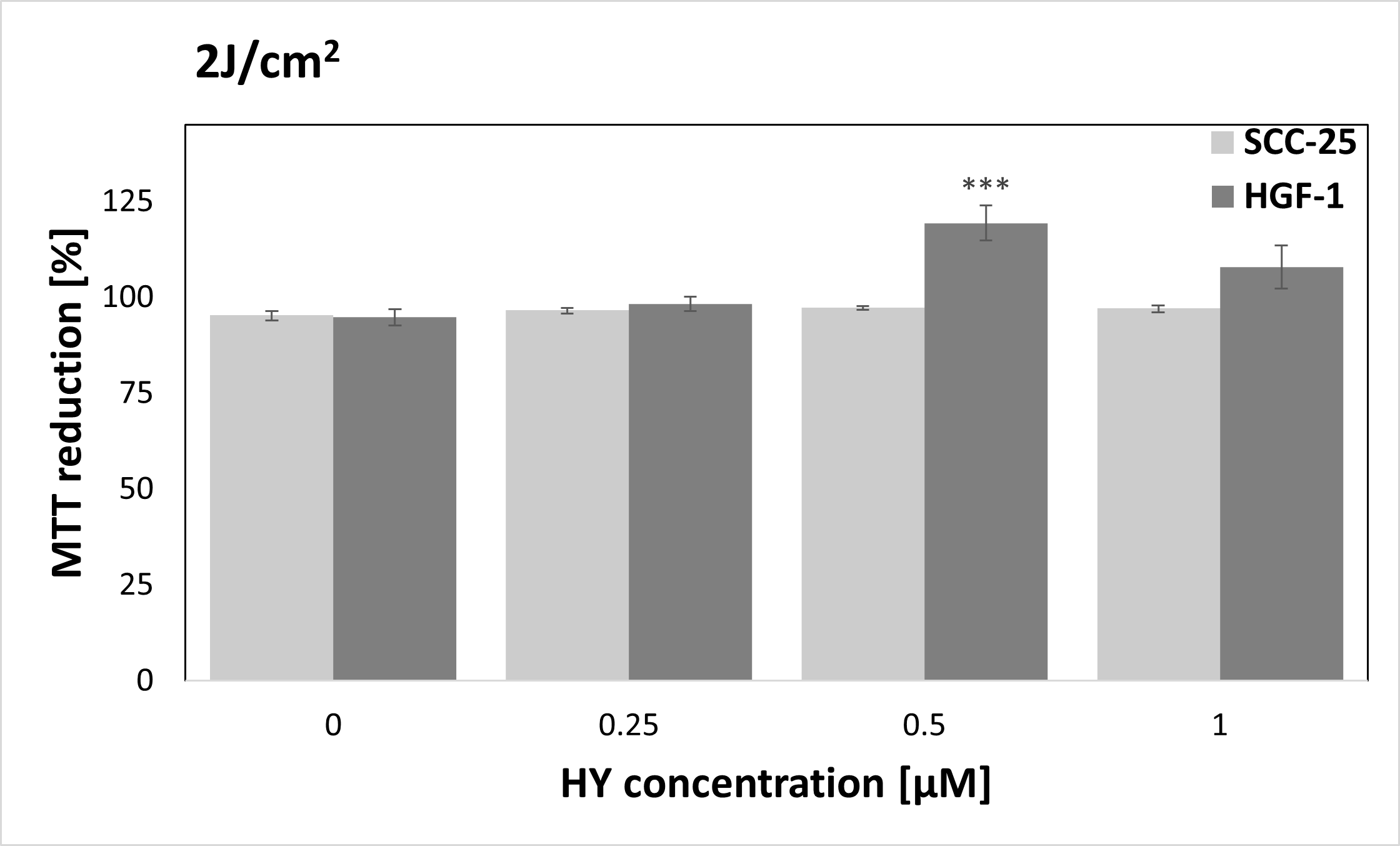

Supplement: Supplementary file 1 [file pharmaceutics-16-00042-s001.zip › Figure S5.png]

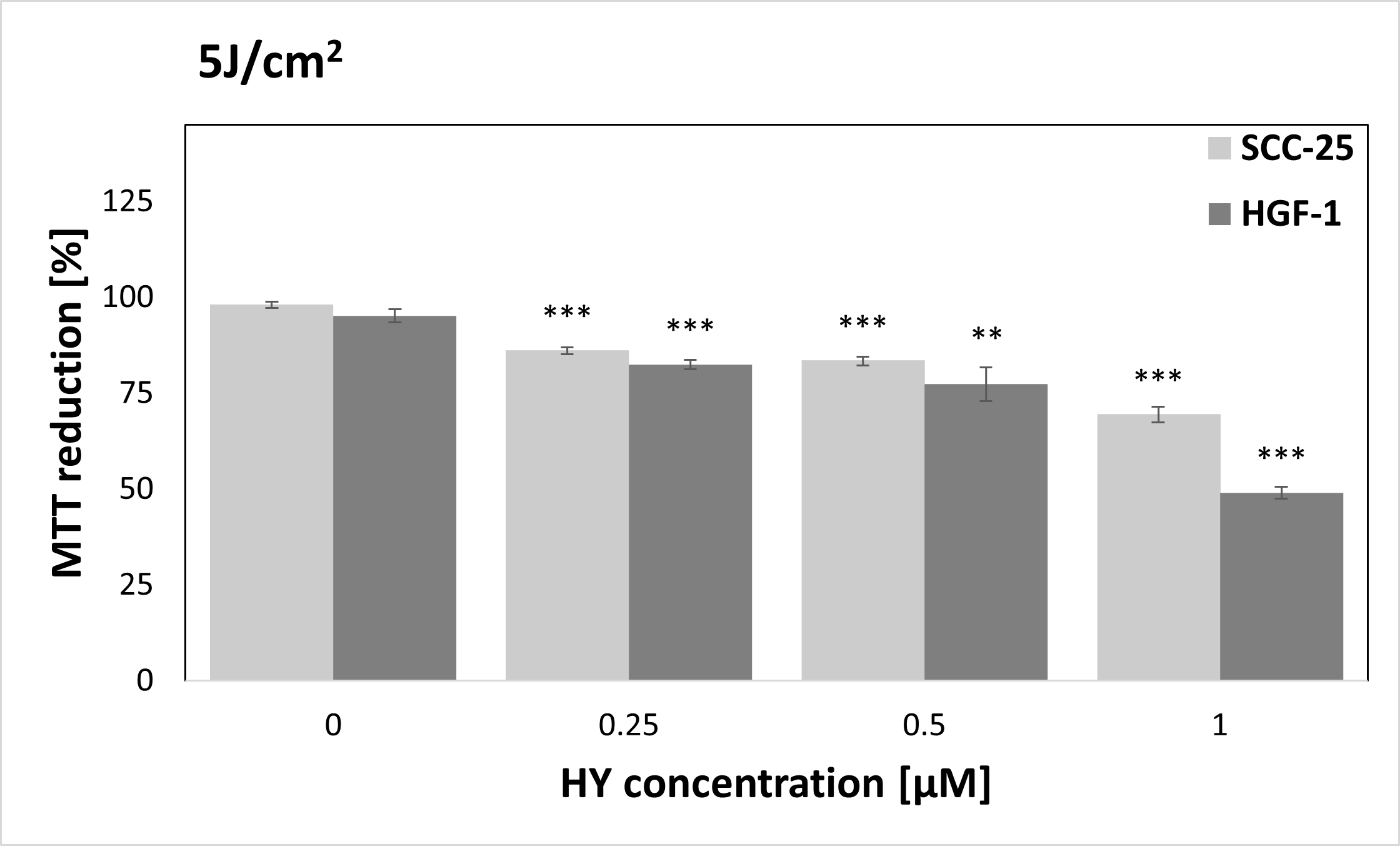

Supplement: Supplementary file 1 [file pharmaceutics-16-00042-s001.zip › Figure S6.png]

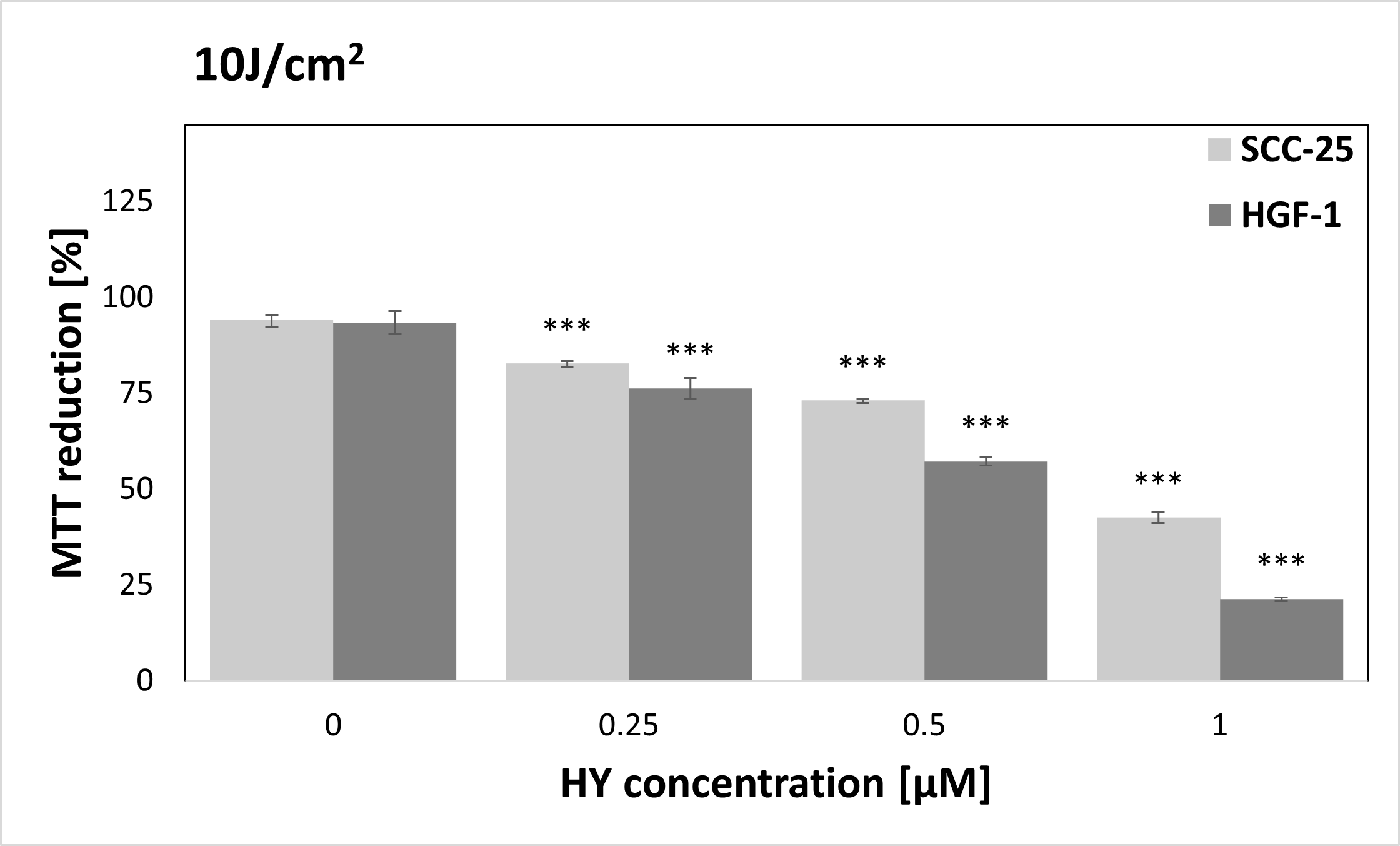

Supplement: Supplementary file 1 [file pharmaceutics-16-00042-s001.zip › Figure S7.png]
